# Supplementary material for: Splice-Junction-Based Mapping of Alternative Isoforms in the Human Proteome
Source: Cell Rep. Author manuscript; Available in PMC 2020 Jan 15. (PMC6961840; doi:10.1016/j.celrep.2019.11.026)
Supplement: 2 [file NIHMS1546469-supplement-2.zip › DF1/PXD009737/TestisLysC/TestisLysC_30_DENND4C_VLFEMKTARIKPNAITYGYYNKEIK.pdf]

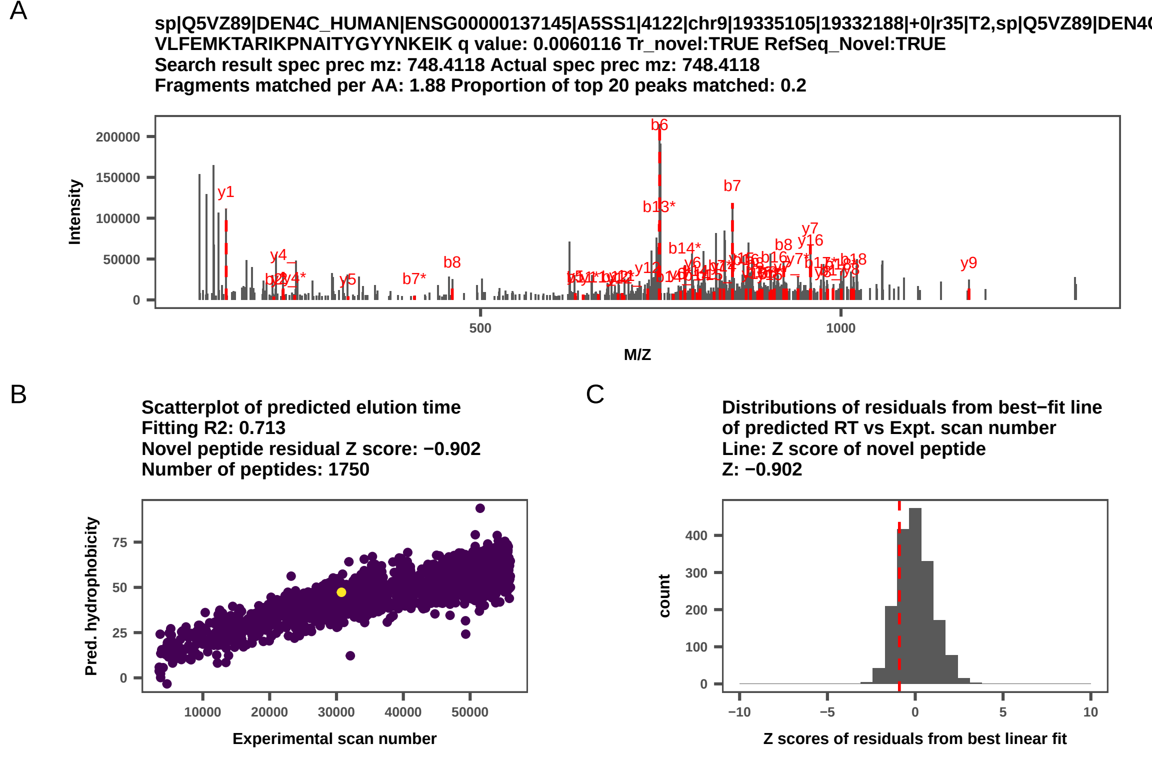
A
sp|Q5VZ89|DEN4C_HUMAN|ENSG00000137145|A5SS1|4122|chr9|19335105|19332188|+0|r35|T2,sp|Q5VZ89|DEN4C
VLFEMKTARIKPNAITYGYYNKEIK q value: 0.0060116 Tr_novel:TRUE RefSeq_Novel:TRUE
Search result spec prec mz: 748.4118 Actual spec prec mz: 748.4118
Fragments matched per AA: 1.88 Proportion of top 20 peaks matched: 0.2
Intensity
200000
150000
100000
50000
0
y1
y4
y5
b7*
b8
b6
b13*
b7
b14*
y7
y16
b8
y12
y9
500
1000
M/Z
B
Scatterplot of predicted elution time
Fitting R2: 0.713
Novel peptide residual Z score: −0.902
Number of peptides: 1750
Pred. hydrophobicity
75
50
25
0
10000
20000
30000
40000
50000
Experimental scan number
C
Distributions of residuals from best−fit line
of predicted RT vs Expt. scan number
Line: Z score of novel peptide
Z: −0.902
count
400
300
200
100
0
−10
−5
0
5
10
Z scores of residuals from best linear fit
